# Curcumol alleviates liver fibrosis by inducing endoplasmic reticulum stress-mediated necroptosis of hepatic stellate cells through Sirt1/NICD pathway

**DOI:** 10.7717/peerj.13376

**Published:** 2022-05-12

**Authors:** Sumin Sun, Sheng Huan, Zhanghao Li, Yue Yao, Ying Su, Siwei Xia, Shijun Wang, Xuefen Xu, Jiangjuan Shao, Zili Zhang, Feng Zhang, Jinbo Fu, Shizhong Zheng

**Affiliations:** 1 Jiangsu Key Laboratory for Pharmacology and Safety Evaluation of Chinese Materia Medica, Nanjing University of Chinese Medicine, Nanjing, China; 2 School of Pharmacy, East China University of Science and Technology, Shanghai, China; 3 Shandong Co-innovation Center of TCM Formula, College of Traditional Chinese Medicine, Shandong University of Traditional Chinese Medicine, Jinan, China; 4 Department of Pharmacy, Jiangsu Province Hospital of Chinese Medicine, Affiliated Hospital of Nanjing University of Chinese Medicine, Nanjing, China

**Keywords:** Curcumol, Necroptosis, ER stress, Sirt1, NICD, Liver fibrosis

## Abstract

Liver fibrosis is a repair response process after chronic liver injury. During this process, activated hepatic stellate cells (HSCs) will migrate to the injury site and secrete extracellular matrix (ECM) to produce fibrous scars. Clearing activated HSCs may be a major strategy for the treatment of liver fibrosis. Curcumol isolated from plants of the genus Curcuma can effectively induce apoptosis of many cancer cells, but whether it can clear activated HSCs remains to be clarified. In the present study, we found that the effect of curcumol in treating liver fibrosis was to clear activated HSCs by inducing necroptosis of HSCs. Receptor-interacting protein kinase 3 (RIP3) silencing could impair necroptosis induced by curcumol. Interestingly, endoplasmic reticulum (ER) stress-induced cellular dysfunction was associated with curcumol-induced cell death. The ER stress inhibitor 4-PBA prevented curcumol-induced ER stress and necroptosis. We proved that ER stress regulated curcumol-induced necroptosis in HSCs *via* Sirtuin-1(Sirt1)/Notch signaling pathway. Sirt1-mediated deacetylation of the intracellular domain of Notch (NICD) led to degradation of NICD, thereby inhibiting Notch signalling pathway to alleviate liver fibrosis. Specific knockdown of Sirt1 by HSCs in male ICR mice further exacerbated CCl_4_-induced liver fibrosis. Overall, our study elucidates the anti-fibrotic effect of curcumol and reveals the underlying mechanism between ER stress and necroptosis.

## Introduction

Liver fibrosis is the repair reaction process after chronic liver injury. Although fibrogenesis and fibrosis represent the attempt to limit the consequences of chronic liver injury in chronic wound healing reaction to some extent, they represent the common pathological process of various chronic liver diseases eventually leading to cirrhosis and liver failure (Parola & Pinzani, 2019; Zhang et al., 2021). Myofibroblasts (MFs), which are not present in the normal liver, are activated in response to liver injury. MFs are the main source of ECM in the fibrotic liver and therefore are the main target for anti-fibrotic therapy (Higashi, Friedman & Hoshida, 2017). In the case of chronic liver injury, HSCs are activated through the process of activation and transdifferentiation into MFs-like cells. HSCs, located in the Disse space, are in close contact with hepatocytes, hepatic sinusoidal endothelial cells, adjacent stellate cells, and nerve terminals through cytoplasmic protrusions, and are physiologic responsible for the synthesis and remodeling of ECM, storage and metabolism of vitamin A and retinoid (Tacke & Trautwein, 2015; Jiang et al., 2021). Activated HSCs are cells that exhibit the unique characteristics of liver MFs, and the elimination of activated HSCs is a strategy to reverse liver fibrosis. Necroptosis is a novel form of programmed cell death dependent on RIP1/RIP3, characterized by plasma membrane disruption, swollen cell lysis and release of damage-associated molecular patterns (Yuan, Amin & Ofengeim, 2019). There are few studies on necroptosis in HSCs, and it is of great significance to investigate the underlying mechanisms.

ER stress refers to the reduced ability of the ER to process intracellular proteins in response to a variety of pathological stimuli, resulting in the accumulation and misfolding of large amounts of proteins in the lumen of the ER, disrupting the original intracellular homeostasis (Chen & Cubillos-Ruiz, 2021; Lebeaupin et al., 2018). In response to changes in the external environment, the ER initiates a series of signaling pathways to process the unfolded and misfolded proteins, which is collectively known as the unfolded protein response (UPR) (Lebeau et al., 2018). Therefore, signature molecules of UPR, such as Glucose regulated protein 78 (GRP78), ER stress effector proteins (PERK, ATF6, iRE-1), are commonly used to indicate the occurrence of ER stress (Mao et al., 2020). However, when ER stress is too severe or lasts too long, UPR is insufficient to restore homeostasis and becomes a toxic signal leading to cell death (Zhang et al., 2018a; Qi et al., 2020). There is increasing evidence that ER stress-induced cellular dysfunction and death are associated with several human diseases, such as neurodegenerative diseases, inflammation, and cancer (Ghemrawi & Khair, 2020; Sprenkle et al., 2017; Li et al., 2019). Whether ER stress is involved in necroptosis of HSCs induced by curcumol deserves further investigation.

The Notch signaling pathway is extensively involved in liver regeneration, injury repair, and metabolic processes, and plays an important role in the transdifferentiation process of HSCs (Xie et al., 2013). The Notch family of transmembrane receptors (Notch1-4) determines cell fate by binding to ligands and γ-secretase mediated cleavage to generate the Notch intracellular domain (NICD), which binds RBPJ-k (recombination signal binding protein for immunoglobulin kappa J) and MAML1 (mastermind-like transcriptional coactivator 1) to activate transcription of target genes such as Hes and Hey family genes, exerting the corresponding biological effects (Jeong et al., 2021). Importantly, Sirt1 is involved in the regulation of Notch signaling pathway. Sirt1 acts as a lysine deacetylase, which regulates the subcellular localisation of NICD by reducing the acetylation level of NICD. The degradation of NICD is also dependent on its hypoacetylation (Marcel et al., 2017; Bai et al., 2018). In recent years, it has been found that Notch signaling pathway is closely related to ER stress, and small-molecule Notch inhibitors can block Notch signaling pathway and induce apoptosis through ER stress pathway (Nolin et al., 2019). Therefore, we have reason to believe that Sirt1/NICD signaling pathway may mediate the bridge between ER stress and necroptosis of HSCs.

Curcumol is isolated from plants of the genus Curcuma, and has many biological activities such as anti-cancer, anti-microbial, and anti-fungal (Wei et al., 2019). Curcumol has great potential to become an anti-cancer drug because it can exert its effects against cancer by inhibiting the cell cycle, inducing cell apoptosis, and regulating various signaling cascades (Li et al., 2018; Liu et al., 2019; Zuo et al., 2020). However, little research has been done on its role in liver fibrosis. In this study, we clarified the ameliorative effects of curcumol on liver fibrosis and tried to reveal the potential link between ER stress and necroptosis.

## Materials and Methods

### Reagents and antibodies

Curcumol (C_15_H_24_O_2_) was obtained from Sigma-Aldrich (St Louis, MO, USA). Dulbecco’s modified essential medium (DMEM), fetal bovine serum (FBS), and trypsin-EDTA were bought from GIBCO BRL (Grand Island, NY, USA). Primary antibodies against GRP78, CHOP, Sirt1, α-SMA, RIP3 were provided from Proteintech Group (Rosemont, IL, USA). Main antibodies against XBP1, RIPK1, ATF4, and COL1A1 were procured from ABclonal Biotechnology Co., Ltd (Wuhan, China). The NICD antibody was obtained by Cell Signaling Technology (Danvers, MA, USA). Z-VAD-FMK and 4-PBA were purchased from MCE (Monmouth Junction, NJ, USA). RO4929097 was provided by APExBIO (Houston, TX, USA). SRT1720 was purchased by Shanghai yuanye Bio-Technology Co., Ltd (Shanghai, China).

### Animal procedures and treatments

All animal care and experimental procedures complied with the National Institutes of Health (Bethesda, MD, USA) guidelines and were approved by the Institutional and Local Committee on the Care and Use of Animals of Nanjing University of Chinese Medicine (approve number: 202009A048). Animal studies were reported in compliance with the ARRIVE guidelines. Forty-four male ICR mice were purchased from Hangzhou Medical College (Hangzhou, China). All mice were maintained in a 12:12 light/dark cycle and provided food and water freely. These mice were randomly divided into six groups (six each in the normal and model groups and eight each in the other groups), using a computer based random order generator. Mice of six groups were treated with olive oil, CCl_4_, CCl_4_+curcumol, CCl_4_+VA-Lip-Sirt1-shRNA, CCl_4_+VA-Lip-Control-shRNA+curcumol, CCl_4_+VA-Lip-Sirt1-shRNA+curcumol, respectively. The mice except the control group were intraperitoneally injected with 0.5 ml/100 g mixture of CCl_4_ and olive oil (1:9 (v/v)) three times a week for 8 weeks. The control group was given the same volume of olive oil. From the fifth week onwards, curcumol was administered intraperitoneally at a dose of 30 mg/kg daily. CCl_4_+VA-Lip-Sirt1-shRNA and CCl_4_+VA-Lip-Control-shRNA were synthesized by Nanjing JinTing biotechnology Co., LTD. and were injected intravenously at a dose of 0.75 mg/kg, 3 times a week for 2 weeks. At the end of the experiment, all mice were anesthetized with pentobarbital (50 mg/kg) and then sacrificed. Part of the liver tissue was taken for histopathology and immunofluorescence studies. The rest of the liver was used to extract protein.

### Histological analysis

The obtained liver tissues were fixed with 4% paraformaldehyde for 12–24 h, and then dehydrated in ethanol at different concentrations, embedded in paraffin, and stained for HE, Masson, Sirius Red and immunohistochemistry, respectively, as needed (Zhang et al., 2018b).

### Cell culture

Human hepatic stellate cells HSC-LX2 (BNCC337957) were purchased from BeNa culture collection (Beijing, China). The cells were cultured in DMEM containing 10% FBS and 1% antibiotics, and incubated at 37 °C in a cell incubator containing 5% CO_2_.

### Trypan blue staining

HSCs were planted in 24-well plates covered with glass slides at appropriate densities, incubated until walled, then test materials were added as needed for the experiment and incubation continued for 24 h. Next, the HSCs were incubated with trypan blue (KeyGEN, #KGY015) for 1–3 min and removed the slides for photographs.

### Cell viability assay

On the basis of previous literature (Lu et al., 2015), we used the MTT method to detect cell viability. The absorbance values at 490 nm was measured using Synergy2 Microplate reader (BioTek, Winooski, VT, USA).

### SiRNA transfection

RIP3 small interfering RNA (siRNA) was synthesized by GenScript (Nanjing, China). Sirt1 siRNA was purchased from KeyGEN (Jiangsu, China). The transfection method was carried out as described earlier (Zhang et al., 2017b).

### Real-time PCR

The total RNA was extracted and qPCR was performed using the Hieff® qPCR SYBR Green Master Mix (YEASEN, #11202ES03). Primer sequences were shown in Table 1. Gadph levels were taken for normalization.

**Table 1:** Primer sequences used in this study.

Gene	Forward sequence	Reverse sequence
chop	TCCAACTGCAGAGATGGCAG	TCCTCCTCTTCCTCCTGAGC
atf6	GCAGAAGGGGAGACACATTT	TTGACATTTTTGGTCTTGTGG
sirt1	GACTCCAAGGCCACGGATAG	TGTTCGAGGATCTGTGCCAA
gadph	ATTCCACCCATGGCAAATTCC	GACTCCACGACGTACTCAGC

### Western blot analysis

After 24 h of incubation with curcumol, proteins were extracted from HSCs. Western blot procedures were then performed according to the manufacturer’s instructions (Bio/Rad, Hercules, CA, USA). The relevant protein bands were quantified using Image Lab software.

### Immunofluorescence analysis

The slides were soaked in 75% alcohol for 30 min, and the residual alcohol was evaporated on an alcohol lamp before placing the slides in a 24-well plate, adding the cell suspension, waiting for the cells to adhere to the wall, then adding test materials and incubating for 24 h. According to previous reports (Lu et al., 2015), immunofluorescence analysis was performed. Briefly, cells on slides were first fixed using 4% paraformaldehyde and left to stand at room temperature for 30 min, followed by permeabilization with 1% Triton-100. Next, the cells were transferred to 1% BSA solution and blocked for 60 min. After blocking, they were incubated with the dilution solution containing the target antibody at 4 °C overnight. The secondary antibody was added and incubated for 2 h at 37 °C in an oven protected from light, then the nucleus were stained with DAPI, and finally the slices were sealed with anti-fluorescence blocking quenching solution, and the fluorescence intensity was observed under a microscope.

### Transmission electron microscopy (TEM)

HSCs were inoculated into 4-well Chambered Coverglass (Thermo Scientific, Waltham, MA, USA) with a cell density of 2 × 10^4^ cells/mL (14,000 cells/well), and then TEM was used to observe the changes in organelle morphology of HSCs after curcumol treatment (Zhang et al., 2018b).

### Co-Immunoprecipitation (Co-IP) experiment

Co-IP was used to observe the interaction between proteins, and the experimental procedures refer to our previously published article (Li et al., 2020).

### Determination of acetylation levels

After the corresponding treatment of the cells, the above IP assay was performed, and then pan-acetylation antibody (Affinity Biosciences, Cincinnati, OH, USA) was used to detect the acetylation level of the NICD.

### Statistical analysis

Both individual cell experiments and animal experiments were performed in duplicate or triplicate, and the matched control group was used to repeat three times, and the data were pooled. All results were expressed as mean ± standard deviation (SD) using the GraphPad Prism 8 (GraphPad software version 8.0). Statistical analysis was performed using either Student’s t-test (two-group comparison) or one-way analysis of variance followed by Tukey’s test (more than two groups). In all analysis, values of *P* < 0.05, *P* < 0.01, and *P* < 0.001 levels were considered significant. N.S., not significant.

## Results

### Curcumol attenuates liver fibrosis by inducing necroptosis of HSCs

**Figure 1:**
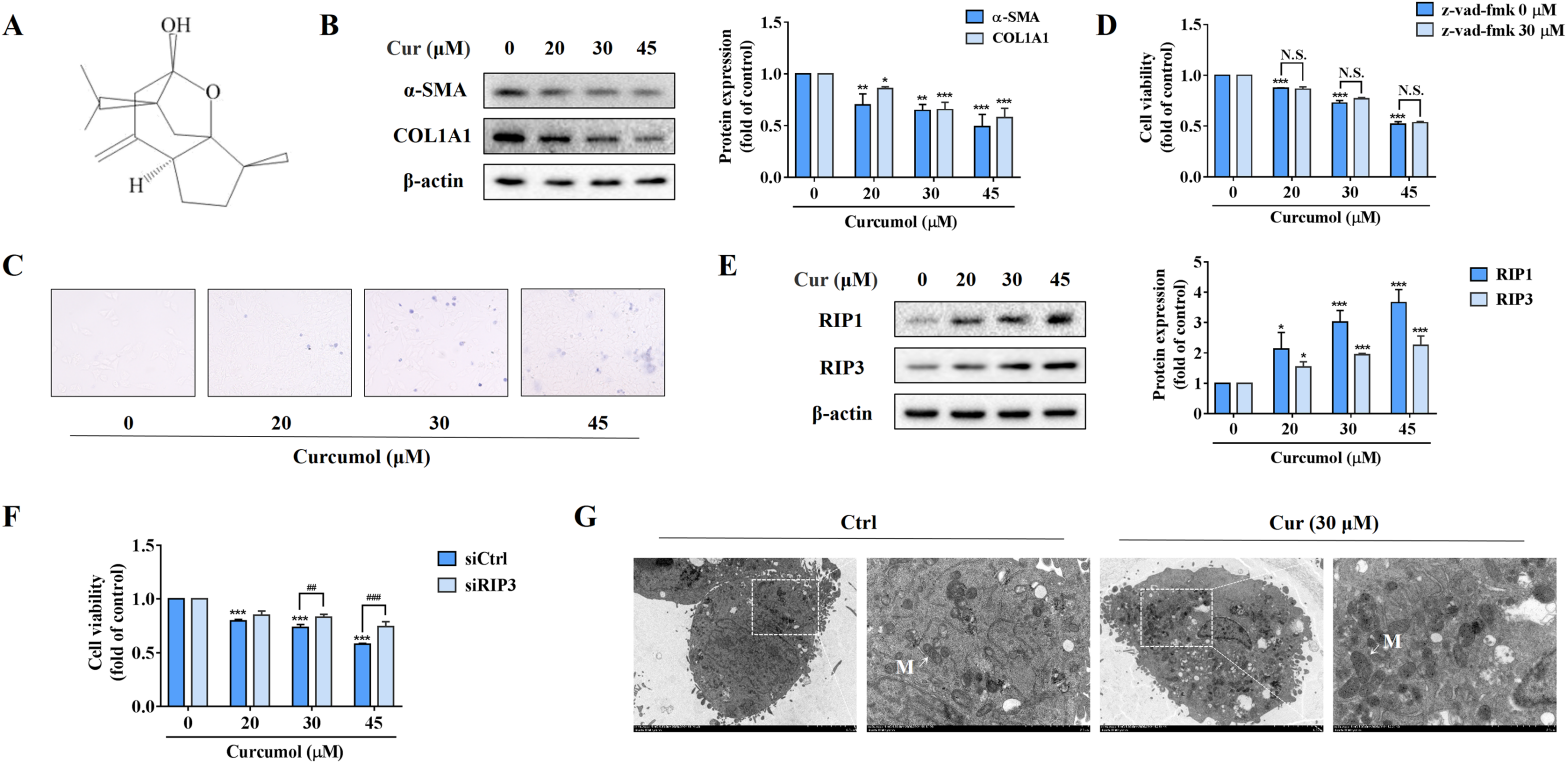
Curcumol attenuates liver fibrosis by inducing necroptosis of HSCs. HSCs were incubated at the specified concentration of curcumol or DMSO for 24 h. (A) Curcumol chemical structure. (B) Protein abundance of α-SMA and COL1A1 were detected. (C) Observing the death of HSCs with trypan blue staining. (D, F) Cell viability was assayed by MTT. (E) The necroptosis-related proteins were analyzed. (G) TEM of HSCs. White arrows indicate mitochondria. Data are expressed as mean ± SD (*n* = 3); *P < 0.05, **P < 0.01, and ***P < 0.001 *vs* DMSO. ^##^*P* < 0.01, ^###^*P* < 0.001 *vs* siCtrl. N.S., not significant.

In order to observe the inhibitory effect of curcumol on HSC-LX2 cells *in vitro*, we first detected the protein abundance of α-SMA and COL1A1 at the specified concentration. Our data fully demonstrated the inhibitory action of curcumol on HSCs activity (Fig. 1B). Furthermore, trypan blue staining showed that curcumol induced HSCs death in a dose-dependent manner (Fig. 1C). Curcumol is involved in the apoptosis process of many cancer cells, but the inhibition of curcumol on HSCs activity was not reversed under the action of apoptosis inhibitor Z-Vad-FMK (Fig. 1D), suggesting that curcumol induced the death of HSCs in a non-apoptotic form. When the apoptotic program fails, necroptosis is often found. Consistently, the experimental results showed that curcumol upregulated RIP1 and RIP3 protein expression in HSCs in a dose-dependent manner (Fig. 1E), and the damaging effect of curcumol on HSCs was significantly weakened after silencing RIP3 (Fig. 1F). Subsequently, TEM also observed that HSCs treated with curcumol exhibited typical necrotic states such as swelling of cells and organelles (Fig. 1G). These results suggested that curcumol reduced liver fibrosis by scavenging activated HSCs through the necroptosis pathway.

### Curcumol induces ER stress in HSCs

**Figure 2:**
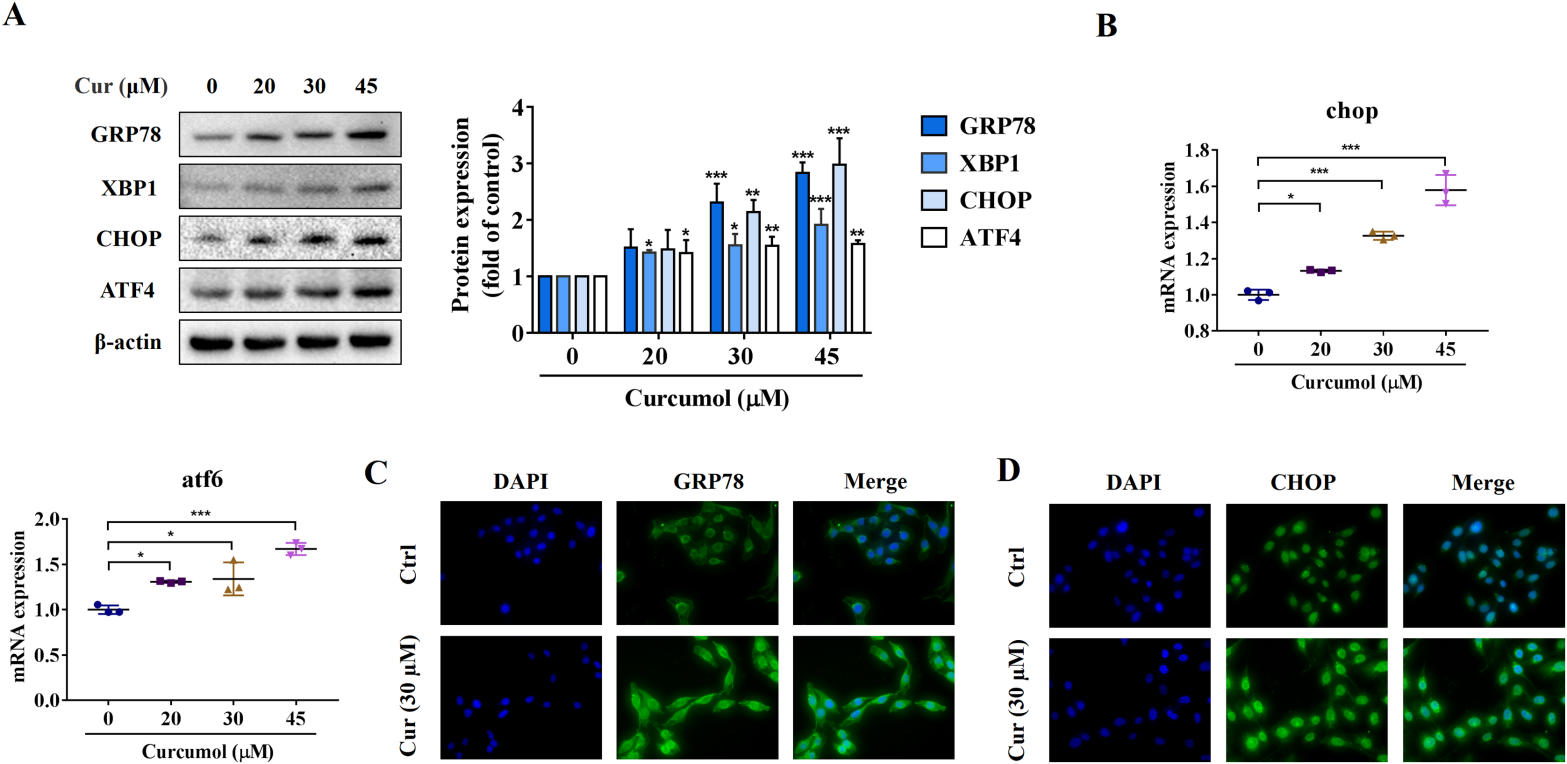
Curcumol induces ER stress in HSCs. (A) The protein abundances of GRP78, XBP1, CHOP and ATF4 were determined after treatment with various concentrations of curcumol for 24 h. (B) ER stress related genes were analyzed. (C, D) The expression of GRP78 (green) and CHOP (green) was analyzed by immunofluorescence assay. Data are expressed as mean ± SD (*n* = 3); *P < 0.05, **P < 0.01, and ***P < 0.001 *vs* DMSO.

ER stress is a signal response pathway system, which belongs to the cell self-protection mechanism. However, if the stress response is too strong or too long, it will cause cell damage (Zhang et al., 2018a; Qi et al., 2020). Whether and how the ER stress is involved in the removal of activated HSCs remains unclear. We therefore investigated the potential mechanisms of curcumol-induced HSCs death by detecting ER stress-related mediators. Western blot analysis showed that the protein abundance of GRP78, X box-binding protein-1 (XBP1), C/EBP-homologous protein (CHOP) and activating transcription factor 4 (ATF4) increased with increasing curcumol dose (Fig. 2A). The transcript levels of chop and atf6 were also enhanced (Fig. 2B). Beyond that, the same results were observed by immunofluorescence (Figs. 2C, 2D). Overall, these results indicated that curcumol activated ER stress, which might be the underlying mechanism for the clearance of activated HSCs.

### ER stress inhibitor impairs the induction effect of curcumol on HSCs necroptosis

**Figure 3:**
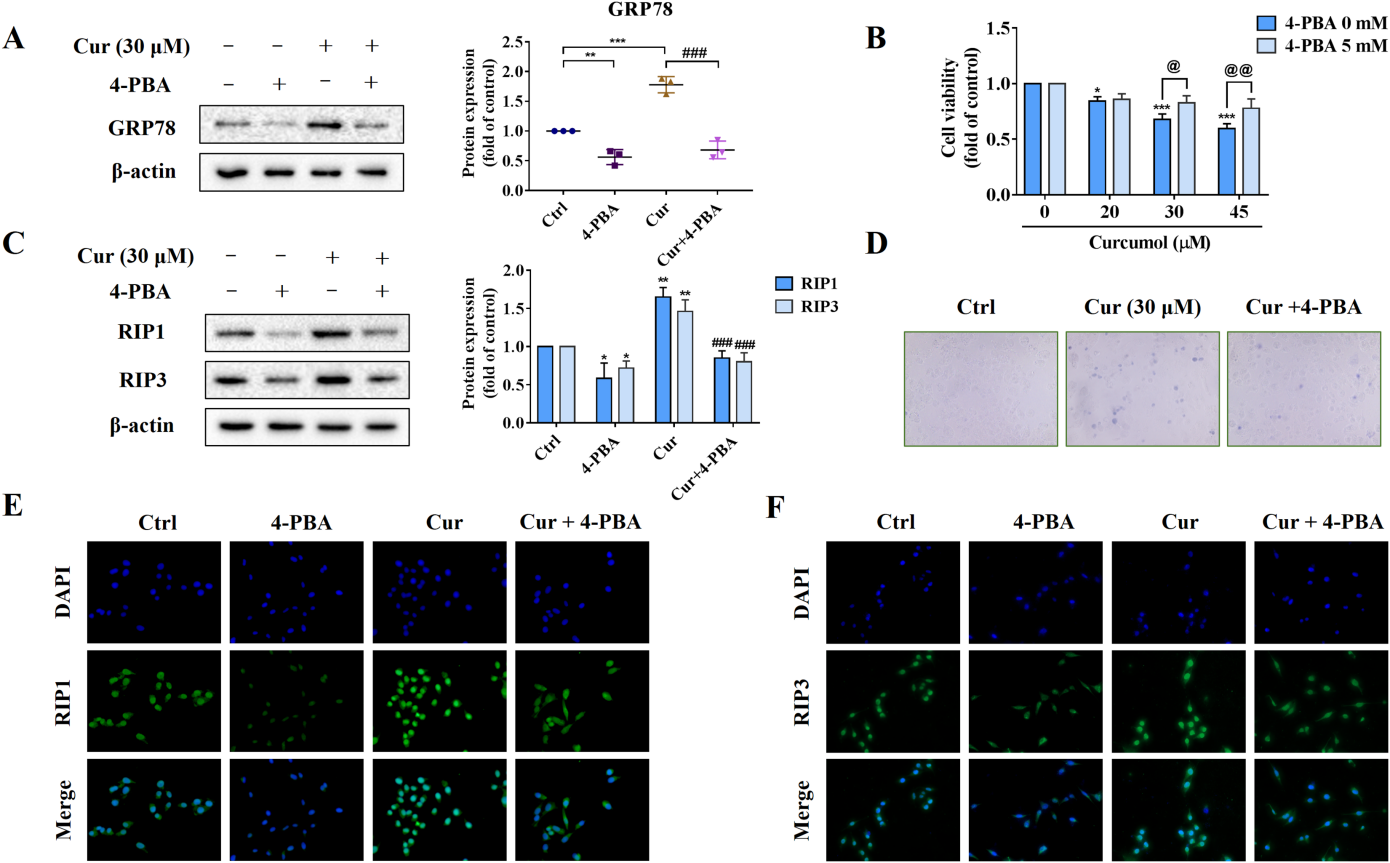
The activation of ER stress may mediate curcumol-induced necroptosis. (A) The inhibitory effect of 4-PBA on ER stress was verified. (B) Cell viability was assayed by MTT. (C) Western blot was used to test the effects of 4-PBA (5 mM) or curcumol (30 µM) on RIP1 and RIP3 in HSCs. (D) Observing the death of HSCs with trypan blue staining. (E, F) Immunofluorescence staining of RIP1 (green) and RIP3 (green). Data are expressed as mean ± SD (*n* = 3); *P < 0.05, **P < 0.01, and ***P < 0.001 *vs* DMSO. ^###^*P* < 0.001 *vs* curcumol. ^@^*P* < 0.05, ^@@^*P* < 0.01 *vs* 4-PBA 0 mM.

To further explore the role of ER stress in curcumol-induced HSCs necroptosis, we used the ER stress inhibitor 4-PBA to block ER stress signalling for reverse validation. First, we verified the inhibitory effect of 4-PBA, and the results showed that 4-PBA could significantly weaken the induction of curcumol on the ER stress marker GRP78 in HSCs (Fig. 3A). Next, we tested the effect of 4-PBA on the viability of HSCs. 4-PBA-mediated ER stress blockage significantly reversed the curcumol-induced HSCs damage (Figs. 3B, 3D). Most importantly, Western blot and immunofluorescence results showed that the promoting effect of curcumol on RIP1 and RIP3 was significantly reduced after 4-PBA intervention (Figs. 3C, 3E, and 3F). In short, these findings supported that the necroptosis induced by curcumol was caused by ER stress.

### ER stress regulates curcumol-induced necroptosis through Sirt1 in HSCs

**Figure 4:**
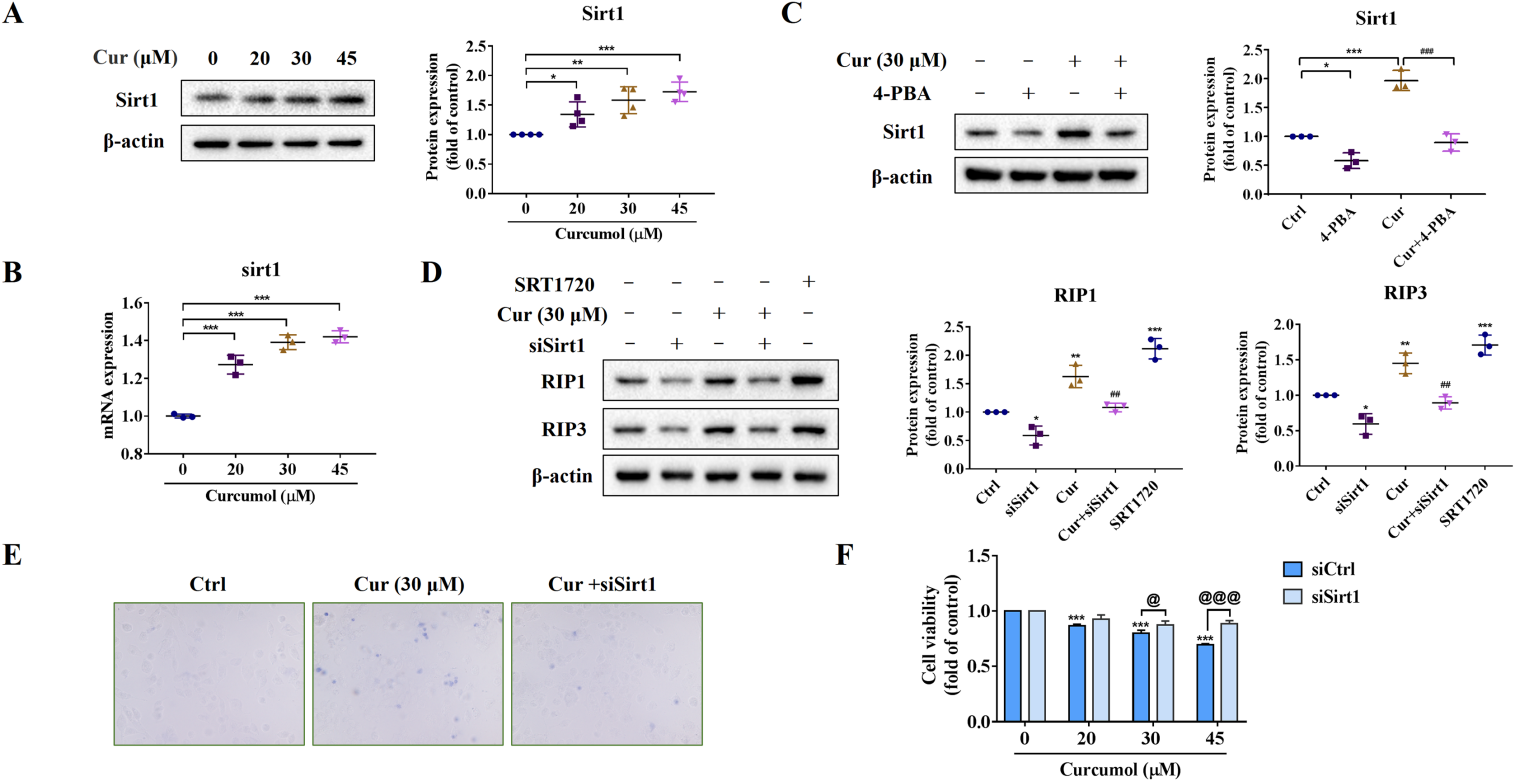
ER stress regulates curcumol-induced necroptosis through Sirt1 in HSCs. (A) The protein abundance of Sirt1 was analyzed. (B) Regulation of Sirt1 at the transcriptional level by curcumol. (C) Western blot was used to test the effect of 4-PBA (5 mM) on Sirt1 in HSCs. (D) The protein expressions of RIP1 and RIP3 after silencing/overexpressing Sirt1 were analyzed. (E) Observing the death of HSCs with trypan blue staining. (F) Cell viability was assayed by MTT. Data are expressed as mean ± SD (*n* = 3); *P < 0.05, **P < 0.01, and ***P < 0.001 *vs* DMSO. ^##^*P* < 0.01, ^###^*P* < 0.001 *vs* curcumol. ^@^*P* < 0.05, ^@@@^*P* < 0.001 *vs* siCtrl.

We further explored the potential mechanism of ER stress promoting necroptosis of HSCs. As an NAD+ dependent class III deacetylase, Sirt1 plays vital physiological function in a variety of diseases such as tumors and metabolic diseases (Alves-Fernandes & Jasiulionis, 2019; Yu et al., 2018), including liver fibrosis (Ramirez et al., 2017). We found that curcumol promoted Sirt1 protein expression (Fig. 4A), and the same results were obtained at the transcription level (Fig. 4B). Importantly, the promotion effect of curcumol on Sirt1 was significantly weakened after 4-PBA treatment (Fig. 4C). Next, we observed the effect of Sirt1 on curcumol-induced necroptosis. Using siSirt1/SRT1720 to silence/stimulate Sirt1 expression, we found that knocksdown of Sirt1 followed by curcumol treatment significantly reduced the protein expression of RIP1 and RIP3 compared to HSCs given curcumol intervention alone, while the Sirt1 agonist SRT1720 exhibited an effect consistent with curcumol, significantly inducing necroptosis in HSCs (Fig. 4D). Finally, trypan blue staining and MTT assay showed that the damaging effect of curcumol on HSCs was dependent on Sirt1 (Figs. 4E and 4F). Collectively, these data showed that the regulation of ER stress on necroptosis in HSCs was closely related to Sirt1.

### Sirt1-mediated deacetylation of NICD is required for curcumol-induced necroptosis in HSCs

**Figure 5:**
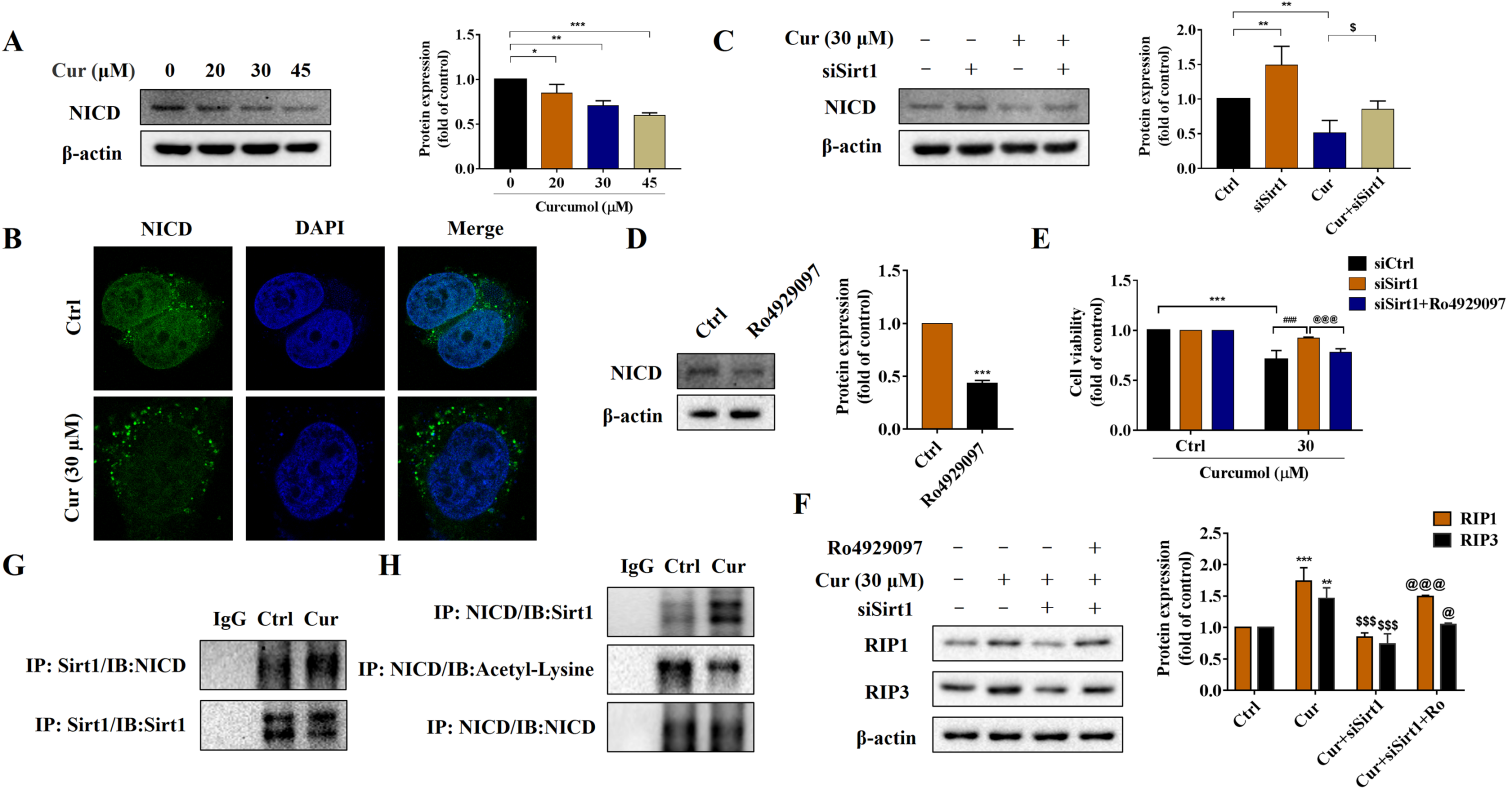
Sirt1-mediated deacetylation of NICD is required for curcumol-induced necroptosis in HSCs. (A) The protein abundance of NICD was analyzed. (B) Immunofluorescence staining of NICD (green). (C) The protein abundances of NICD after Sirt1 knockdown was investigated. (D) The inhibitory effect of γ-secretase inhibitor Ro4929097 (1 µM) on NICD was verified. (E) Cell viability was assayed by MTT. (F) Western blot was used to test the effect of Ro4929097 on RIP1 and RIP3 in HSCs. (G) CO-IP assay detected the interaction between Sirt1 and NICD. (H) The acetylation level of NICD was assayed by IP assay. Data are expressed as mean ± SD (*n* = 3); *P < 0.05, **P < 0.01, and ***P < 0.001 *vs* DMSO. ^$^*P* < 0.05, and ^$$$^*P* < 0.001 *vs* curcumol. ^###^*P* < 0.001 *vs* Cur+siCtrl. ^@^*P* < 0.05, ^@@@^*P* < 0.001 *vs* Cur+siSirt1.

Notch signaling pathway plays an important role in liver fibrosis (Xie et al., 2013). Under the intervention of curcumol, the expression level of NICD decreased in a dose-dependent manner (Fig. 5A), and laser confocal observations showed that curcumol significantly inhibited the entry of NICD into the nucleus (Fig. 5B). Next, we looked at whether this effect of curcumol was associated with Sirt1. Sirt1 silencing mediated by siRNA significantly reversed curcumol induced NICD reduction (Fig. 5C). The molecular mechanism of curcumol damage to HSCs was further validated by reducing NICD protein abundance with the γ-secretase inhibitor Ro4929097 (Fig. 5D). MTT assay showed that silencing Sirt1-induced upregulation of HSCs activity was significantly reversed by intervention with Ro4929097 (Fig. 5E). Western blot assay further showed that Sirt1 silencing impaired curcumol induced necroptosis of HSCs, and Ro4929097 further reversed this effect (Fig. 5F). These experimental results suggested that curcumol might regulate necroptosis of HSCs through Sirt1/NICD signaling pathway. Next, we conducted an in-depth study on how Sirt1 regulates NICD. Co-IP results showed that the interaction between Sirt1 and NICD was enhanced in the presence of curcumol, and the acetylation level of NICD was significantly reduced (Figs. 5G, 5H), suggesting that Sirt1 might promote the degradation of NICD through deacetylation, thereby promoting the necroptosis of HSCs.

### Curcumol alleviates liver fibrosis through Sirt1 *in vivo*

**Figure 6:**
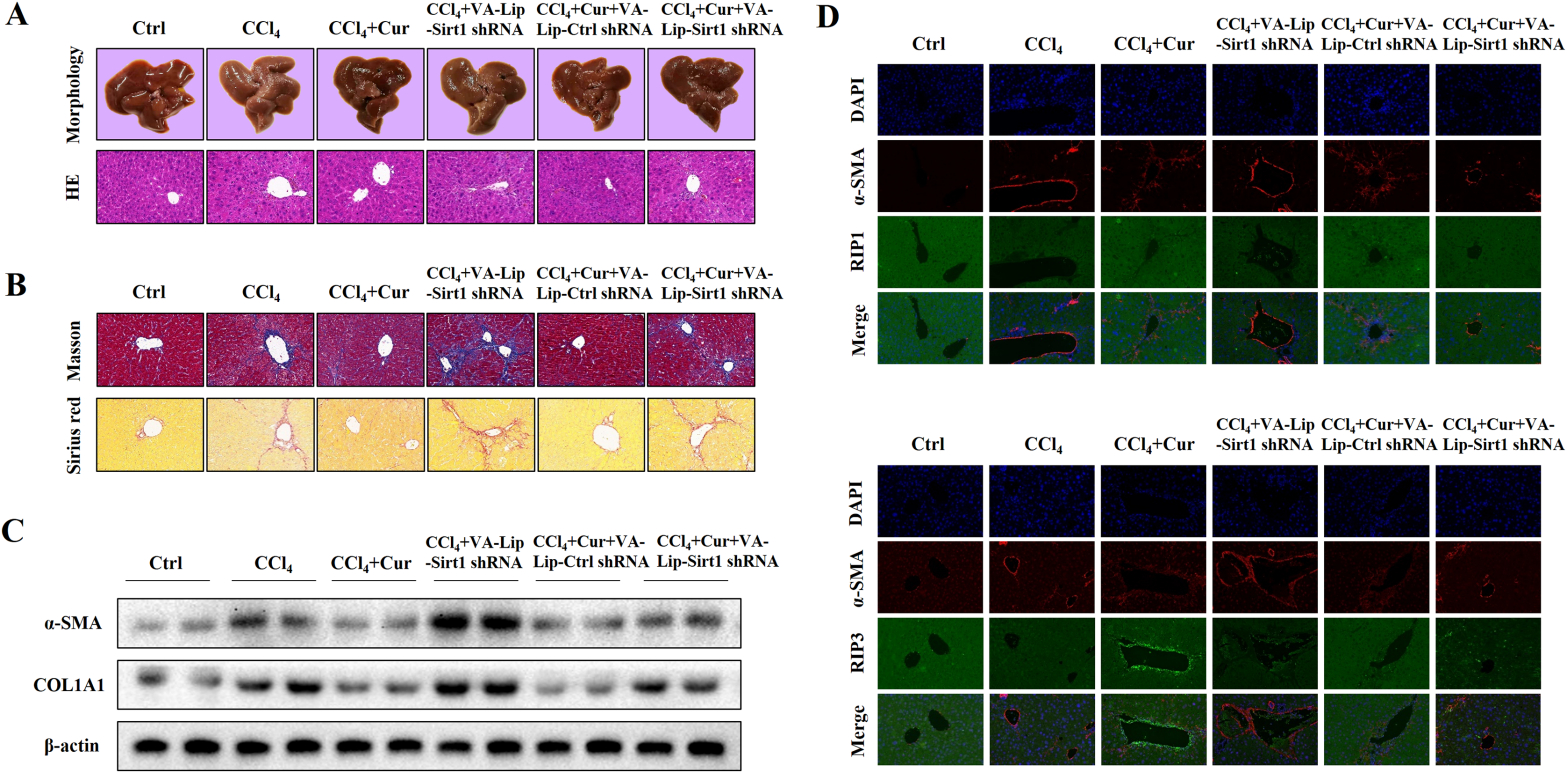
Curcumol reduces liver fibrosis through Sirt1-mediated necroptosis *in vivo*. (A) The morphology and HE staining of mouse livers were analyzed. (B) Analysis of collagen deposition in mouse livers by Masson and Sirius red staining. (C) The expression of α-SMA and COL1A1 in liver tissues. (D, E) The expression and co-localization of α-SMA (red) with RIP1 (green) and RIP3 (green) were determined by immunofluorescence double staining.

To further investigate the effects of curcumol on liver injury and liver fibrosis, we established a classical mouse model of liver fibrosis by intraperitoneal injection of CCl_4_ to observe the therapeutic effects of curcumol. We first determined whether curcumol could ameliorate liver injury *in vivo*. The liver index (liver weight/body weight) was positively correlated with the degree of liver injury (Li et al., 2020). Compared with the normal group, the liver index of mice in the model group was significantly higher, while the liver index of mice in the curcumol group was significantly lower than that of mice in the model group (Fig. S1A). Through morphological observation, we found that the livers of mice in the control group was bright red and shiny with no histological lesions, while the livers of model mice had obvious fibrotic lesions. It was noteworthy that the improvement effect of curcumol on liver fibrosis in mice was mediated by Sirt1. HSCs specific knockout of Sirt1 impaired the ameliorative effect of curcumol on hepatic fibrosis in mice (Fig. 6A). Subsequently, histological examination was performed on the livers of each group of mice. Inflammatory cell infiltration and collagen deposition induced by CCl_4_ were significantly alleviated by curcumol, and this effect was significantly reversed when Sirt1 was silenced (Figs. 6A, 6B and Fig. S1B). In addition, Western blot analysis showed that curcumol could effectively inhibit the expression of α-SMA and COL1A1 in HSCs, and this effect depended on the upregulation of Sirt1 (Fig. 6C).

To clarify the role of necroptosis in the treatment of liver fibrosis with curcumol, we performed double fluorescence staining of liver tissues to label HSCs with α-SMA and detected the expression of RIP1 and RIP3. The results showed that RIP1 and RIP3 co-localized with α-SMA, and the promotion of RIP1 and RIP3 by curcumol was mediated by Sirt1 (Fig. 6D). All together, these results indicated that the ameliorative effect of curcumol on liver fibrosis in mice depended on Sirt1-mediated necroptosis.

## Discussion

Liver fibrosis is a dynamic and highly integrated molecular, cellular, and tissue process characterized by excessive deposition of ECM components, which are mainly derived from hepatic MFs (Schuppan et al., 2018). In chronic liver injury, HSCs will activate and transdifferentiate into MF-like cells, breaking the balance between ECM production and degradation. Therefore, inducing the death of activated HSCs or returning them to a resting state by drug-based interventions is an effective strategy to alleviate liver fibrosis (Higashi, Friedman & Hoshida, 2017). Curcumol is a kind of bioactive sesquiterpenoid isolated from Zingiberaceae plants, which can induce apoptosis of a variety of cancer cells (Wei et al., 2019). However, its role in liver fibrosis is rarely studied, and whether it can alleviate liver injury by removing MF-like HSCs remains to be revealed. Our study showed that curcumol could reduce liver fibrosis by promoting necroptosis of HSCs, and this process was associated with over-activated ER stress.

Because HSCs are rich in ER and secreted proteins, they may be more sensitive to changes in ER homeostasis. ER is an evolutionarily conserved response to dysfunction of the ER caused by genetic and environmental damage that can lead to cell death (Hu et al., 2019). The markers of ER stress may be up-regulation of some related genes, such as ATF6, CHOP, GRP78, etc. (Bian et al., 2019). ER plays a critical role in cell clearance mechanisms, which may be mediated by death receptor and mitochondrial pathways. The classic apoptosis is induced by mitochondria and death receptor, so ER stress has been the focus of the new apoptosis mechanism (Bian et al., 2019). Necroptosis and apoptosis share some upstream signals. Recent studies have shown that necroptosis may be another downstream cell death mode of ER stress. Chang et al. (2019) showed that in the myocardial infarction disease model, down-regulation of the RIP3-CaMKII signaling pathway can alleviate ER stress-related necroptosis and play a role in protecting the heart. Kishino et al. (2019) demonstrated that ER stress can induce either apoptosis of auditory cells or necroptosis. However, the potential relationship between ER stress and necroptosis has not been confirmed in HSCs. In our study, we evaluated the ameliorative effects of curcumol on liver injury and liver fibrosis *in vivo* and *in vitro*, and found that curcumol reduced CCl_4_-induced liver fibrosis *in vivo* and significantly inhibited HSCs cell viability *in vitro*. The damage of curcumol on HSCs depended on the occurrence of necroptosis of HSCs, and this effect was caused by ER stress. Under the action of ER stress inhibitor 4-PBA, the induction effect of curcumol on the necrosome markers RIP1 and RIP3 was significantly reversed. These results suggested that targeting the induction of ER stress and necroptosis of HSCs might be an effective strategy for the treatment of liver fibrosis.

Next, we explored the potential mechanism of curcumol in regulating ER stress and necroptosis. Sirt1 is an NAD+-dependent type III histone deacetylase, which can catalyze the deacetylation of histone and non-histone lysine residues, and plays a vital role in regulating cell apoptosis (Zhang et al., 2017a), the occurrence and development of tumors (Alves-Fernandes & Jasiulionis, 2019), metabolic diseases (Yu et al., 2018) and anti-aging (Xu et al., 2020). Curcumol promoted the expression of Sirt1 in HSCs in a dose-dependent manner, and the effect of curcumol was significantly weakened after 4-PBA treatment. Importantly, we demonstrated the regulatory effect of Sirt1 on necroptosis. After silencing of Sirt1, the induction of necroptosis by curcumol was significantly attenuated, while the expression levels of RIP1 and RIP3 were significantly increased in HSCs in response to the Sirt1 agonist SRT1720. Notch signaling is involved in liver regeneration and repair, liver fibrosis and metabolic processes. Due to these extensive and important roles, the role of Notch signaling in liver development and disease progression and the regulatory mechanisms of this pathway in the liver have been the subject of research in recent years (Xie et al., 2013). Our experimental results showed that Sirt1 could interact with the Notch intracellular domain NICD in HSCs, and promoted its degradation by deacetylating the NICD, thereby inhibiting the Notch signaling pathway. From this, we conclude that ER stress regulates the necroptosis of HSCs induced by curcumol through Sirt1/Notch pathway.

## Conclusions

We reported for the first time the potential connection between ER stress and necroptosis in HSCs, and further clarified that curcumol exerted its anti-fibrotic effects through the Sirt1/Notch signalling pathway, providing a new target and perspective for the treatment of liver fibrosis and the development of related drugs.

## Supplemental Information

10.7717/peerj.13376/supp-1 Supplemental Information 1 Curcumol reduces liver fibrosis through Sirt1-mediated necroptosis *in vivo*. (A) The liver index (liver weight/body weight) of mice. (B) Immunohistochemical staining of α-SMA.

10.7717/peerj.13376/supp-2 Supplemental Information 2 Curcumol alleviates liver fibrosis by inducing endoplasmic reticulum stress-mediated necroptosis of hepatic stellate cells through Sirt1/NICD pathway. Curcumol induces ER stress in HSCs, and ER stress-induced cellular dysfunction causes necroptosis in HSCs. The specific mechanism is through activation of the downstream Sirt1/Notch signaling pathway. Sirt1-mediated deacetylation of the intracellular domain of Notch (NICD) and promotes NICD degradation. The inhibition of Notch signaling pathway contributes to hepatic stellate cell necroptosis and alleviates liver fibrosis.

10.7717/peerj.13376/supp-3 Supplemental Information 3 Repeated results of all corresponding Western Blot results.

10.7717/peerj.13376/supp-4 Supplemental Information 4 The Corresponding qRT-PCR and MTT results.

10.7717/peerj.13376/supp-5 Supplemental Information 5 Checklist.

## Abbreviations

HSCs: Hepatic stellate cells
ECM: Extracellular matrix
RIP: Receptor-interacting protein kinase
α-SMA: α-smooth muscle actin
COL1A1: Collagen type I alpha 1 chain
Sirt1: Sirtuin 1
UPR: Unfolded protein response
GRP78: Glucose regulated protein 78
ATF6: Activating transcription factor 6
CHOP: C/EBP-homologous protein
XBP1: X box-binding protein-1
ER: Endoplasmic reticulum

## Additional Information and Declarations

### Data Availability

The following information was supplied regarding data availability: The raw measurements are available in the Supplemental Files.
