# Supplementary figures and images for: Curcumol alleviates liver fibrosis by inducing endoplasmic reticulum stress-mediated necroptosis of hepatic stellate cells through Sirt1/NICD pathway
Source: PeerJ. 2022 May 12;10:e13376. doi: 10.7717/peerj.13376 (PMC9107784; doi:10.7717/peerj.13376)

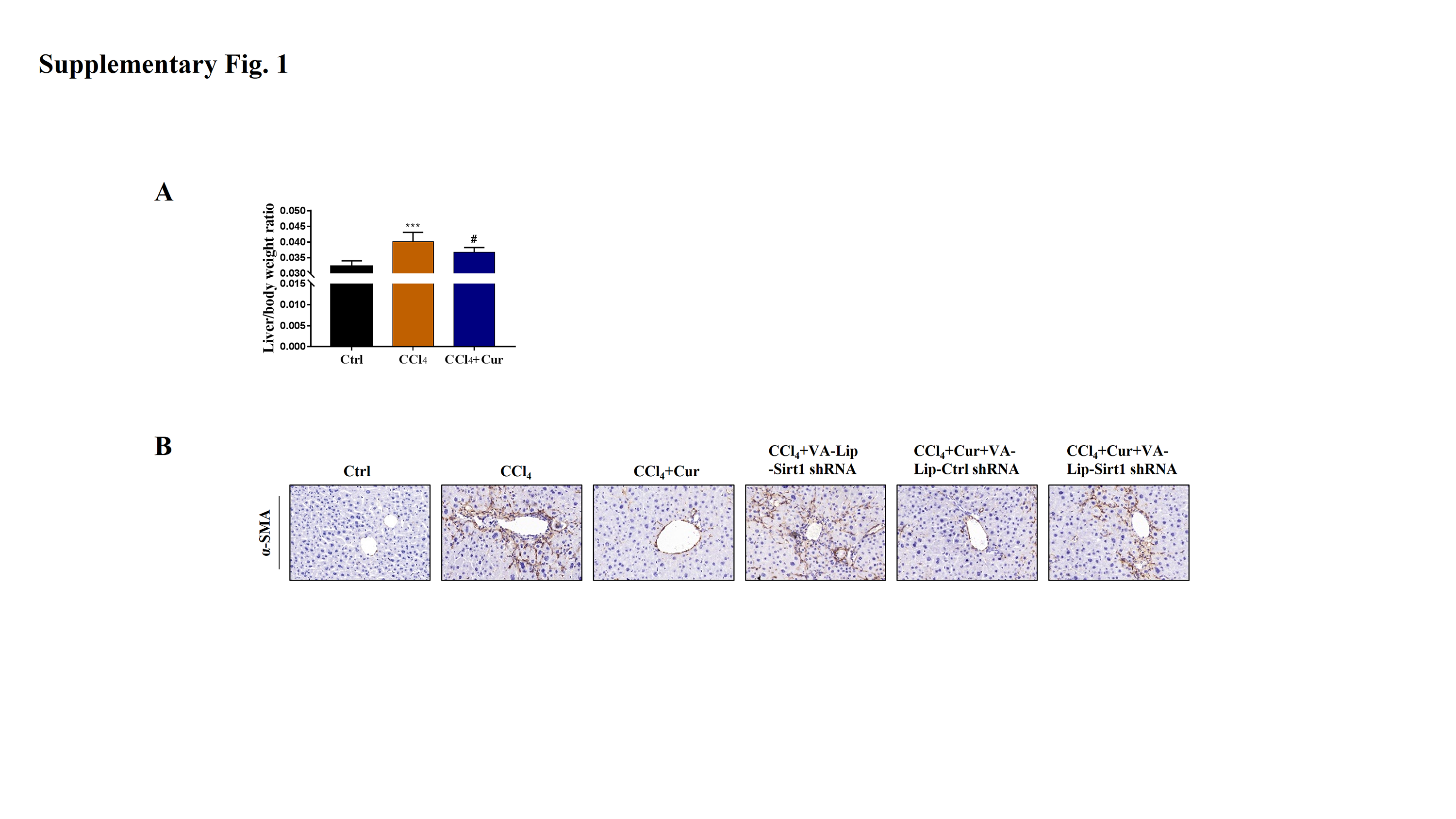

Supplement: Supplemental Information 1 — (A) The liver index (liver weight/body weight) of mice. (B) Immunohistochemical staining of α-SMA. [file peerj-10-13376-s001.png]

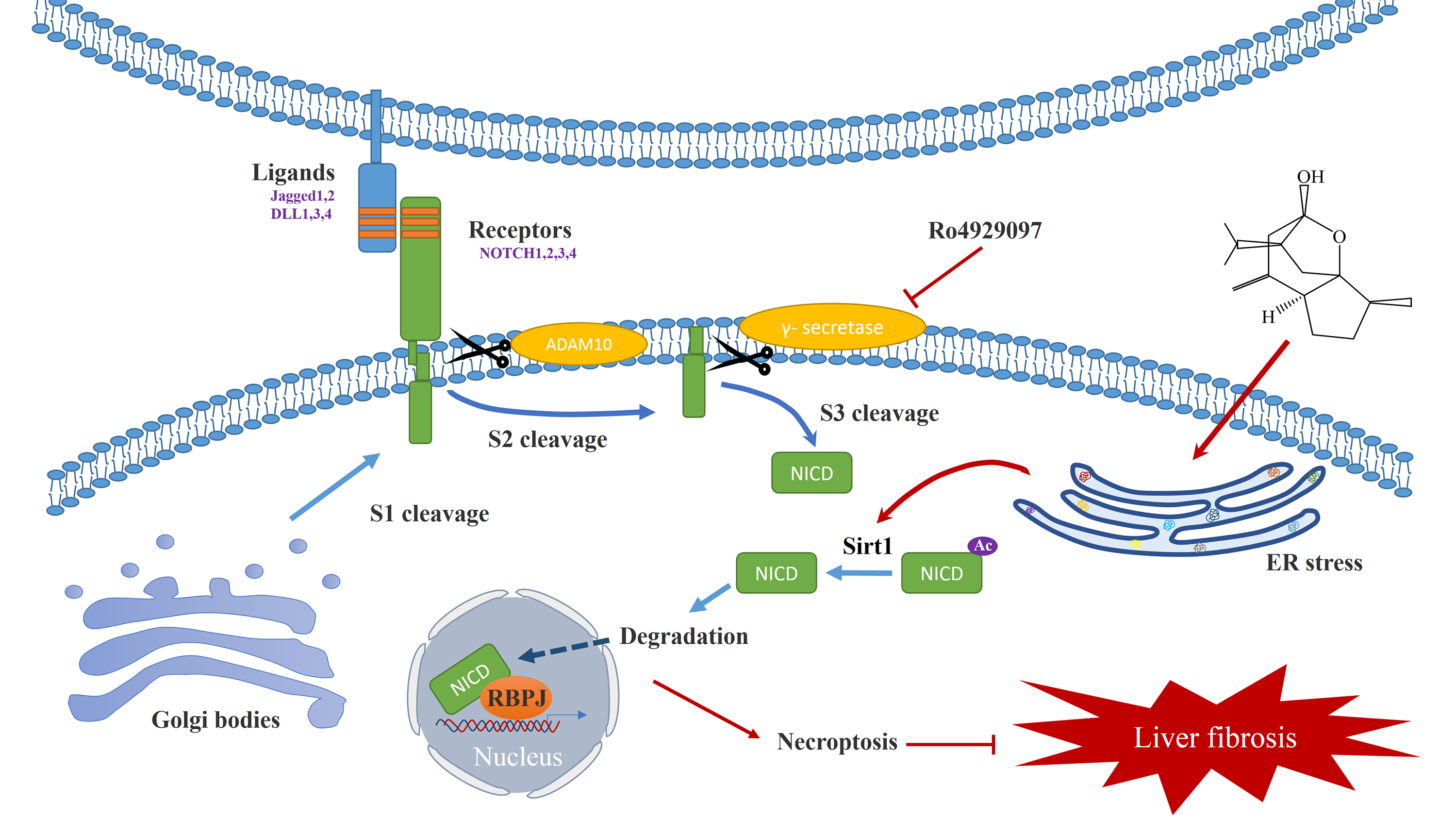

Supplement: Supplemental Information 2 — Curcumol induces ER stress in HSCs, and ER stress-induced cellular dysfunction causes necroptosis in HSCs. The specific mechanism is through activation of the downstream Sirt1/Notch signaling pathway. Sirt1-mediated deacetylation of the intracellular domain of Notch (NICD) and promotes NICD degradation. The inhibition of Notch signaling pathway contributes to hepatic stellate cell necroptosis and alleviates liver fibrosis. [file peerj-10-13376-s002.png]
