# Supplementary material for: Curcumol alleviates liver fibrosis by inducing endoplasmic reticulum stress-mediated necroptosis of hepatic stellate cells through Sirt1/NICD pathway
Source: PeerJ. 2022 May 12;10:e13376. doi: 10.7717/peerj.13376 (PMC9107784; doi:10.7717/peerj.13376)
Supplement: Supplemental Information 3 [file peerj-10-13376-s003.pdf]

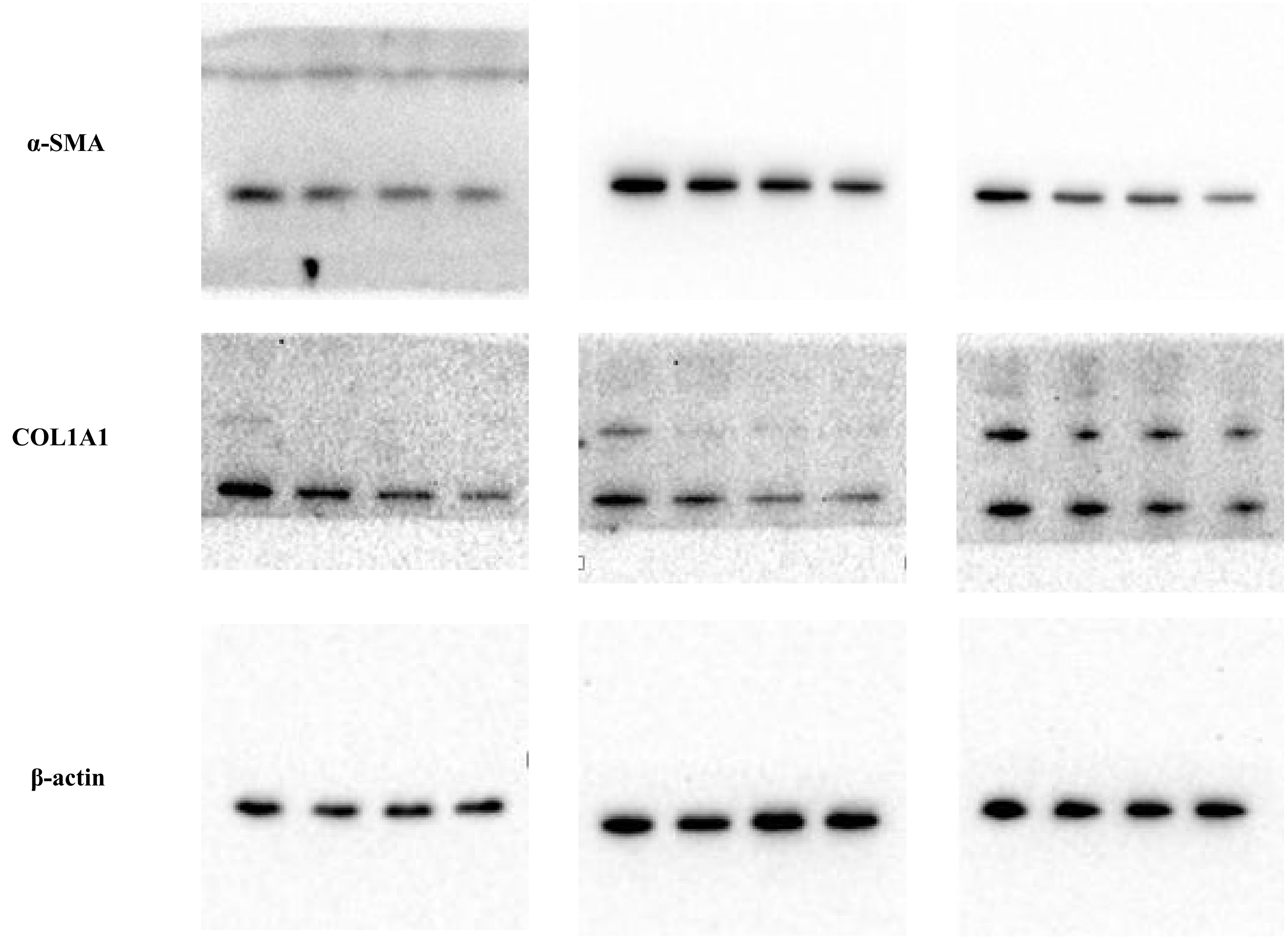
α-SMA
COL1A1
β-actin

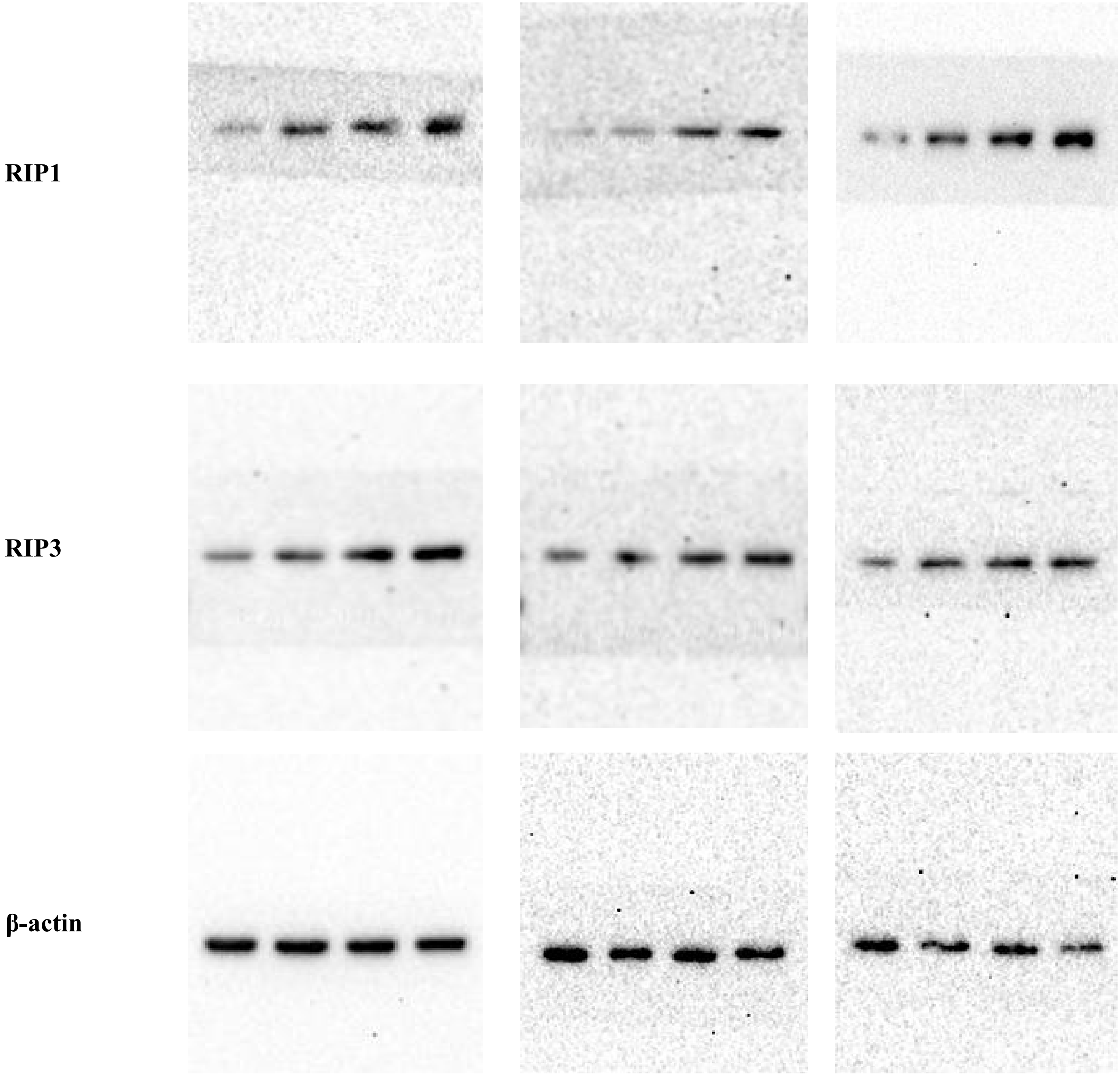
RIP1
RIP3
β-actin

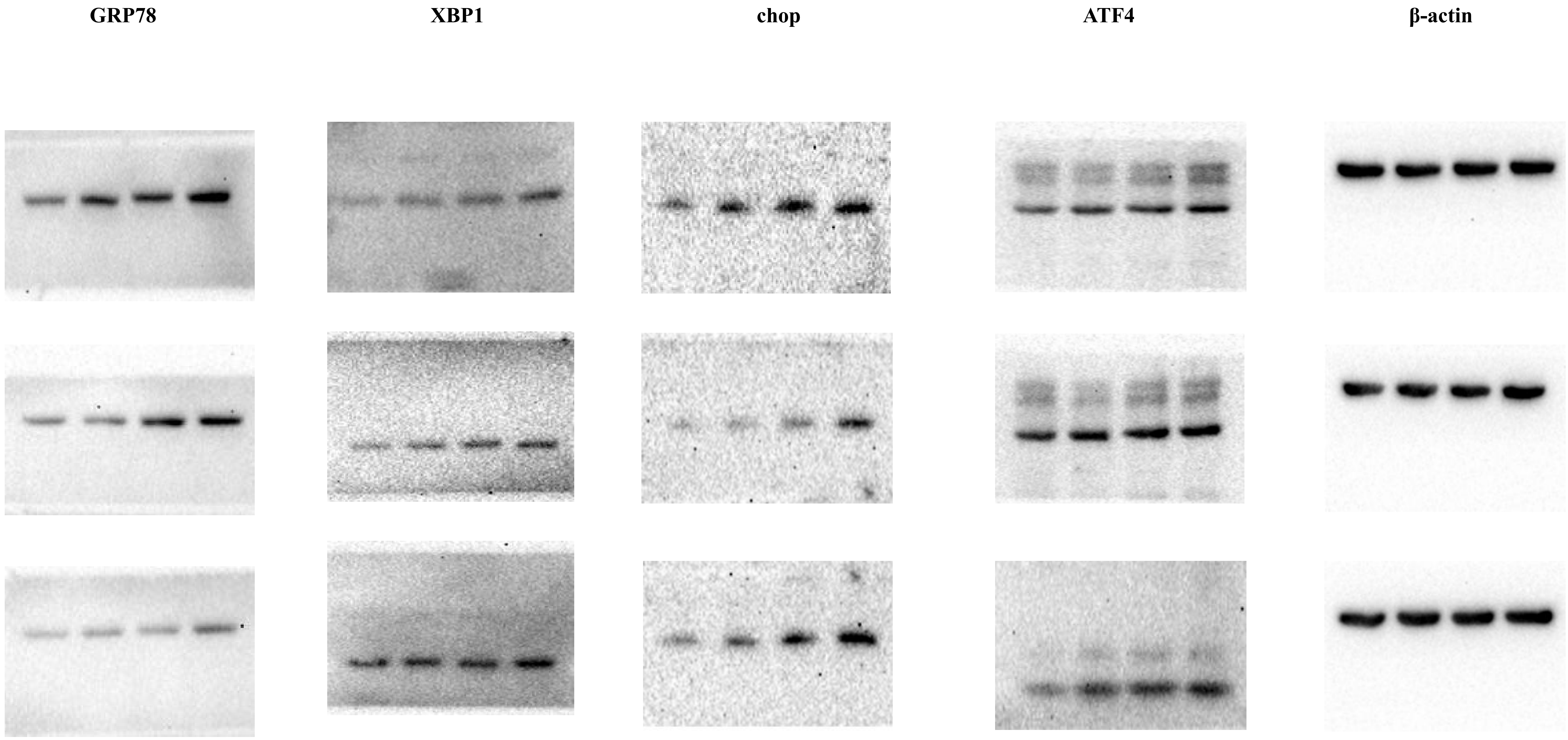
GRP78
XBP1
chop
ATF4
β-actin

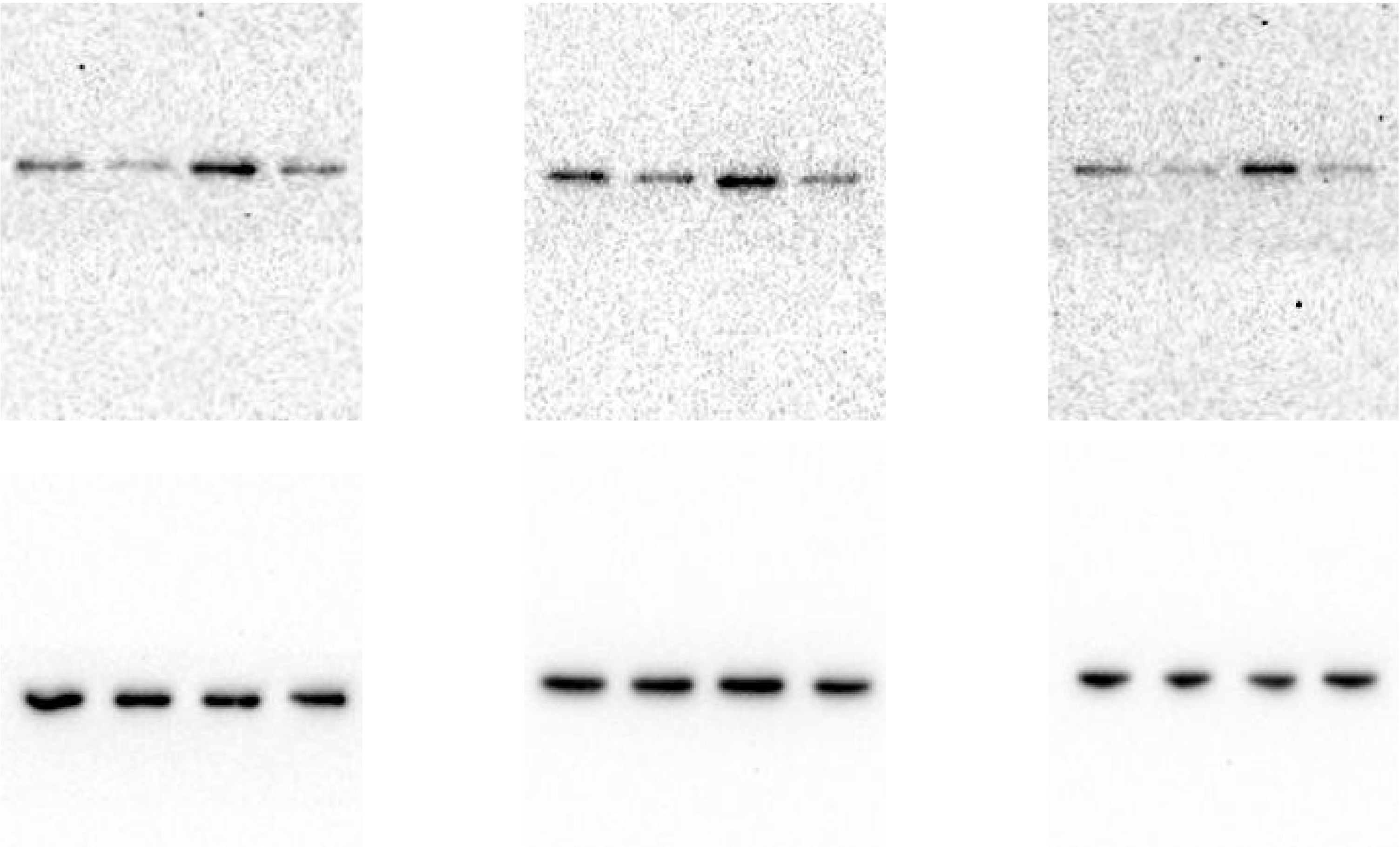
GRP78
β-actin

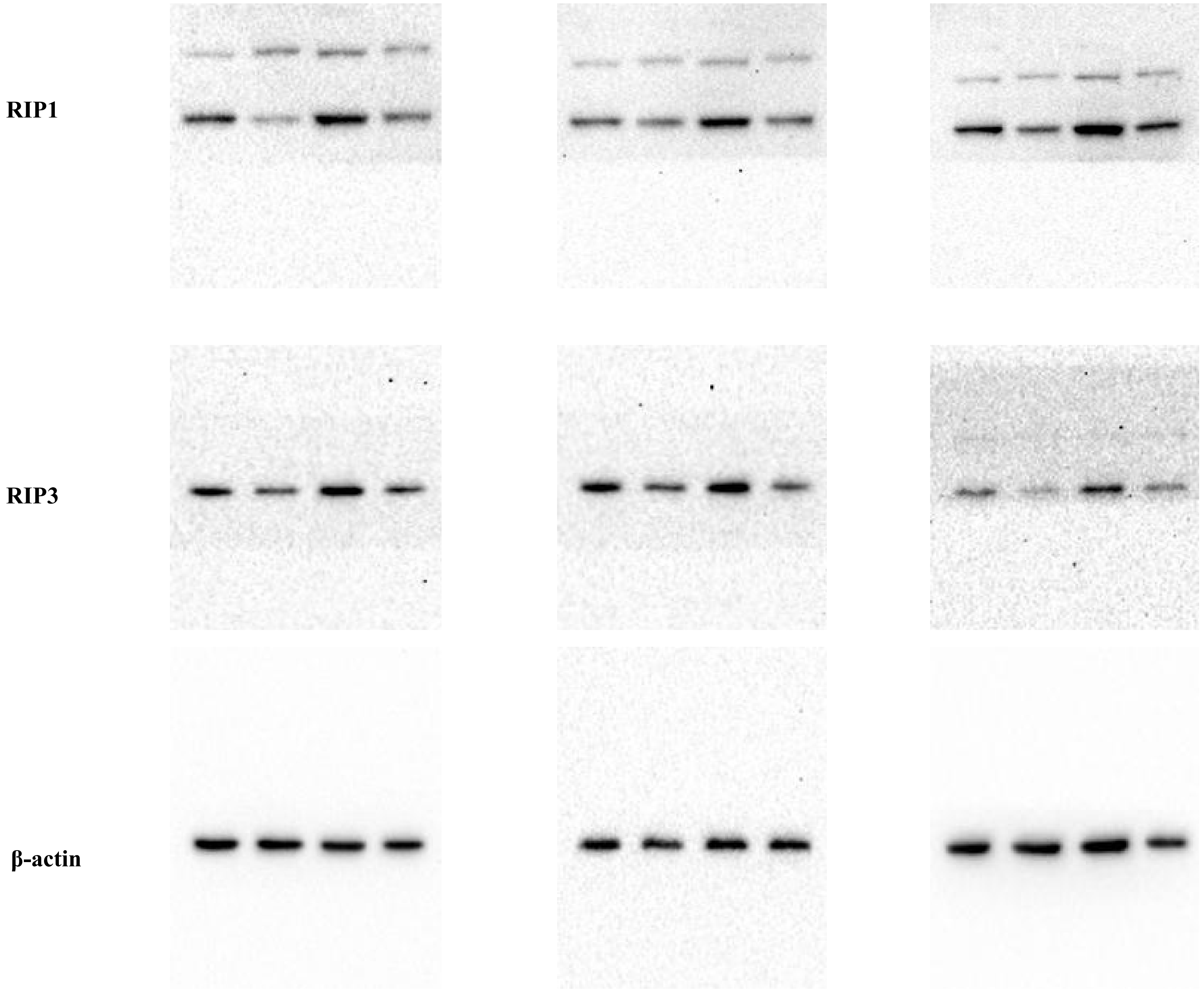
RIP1
RIP3
β-actin

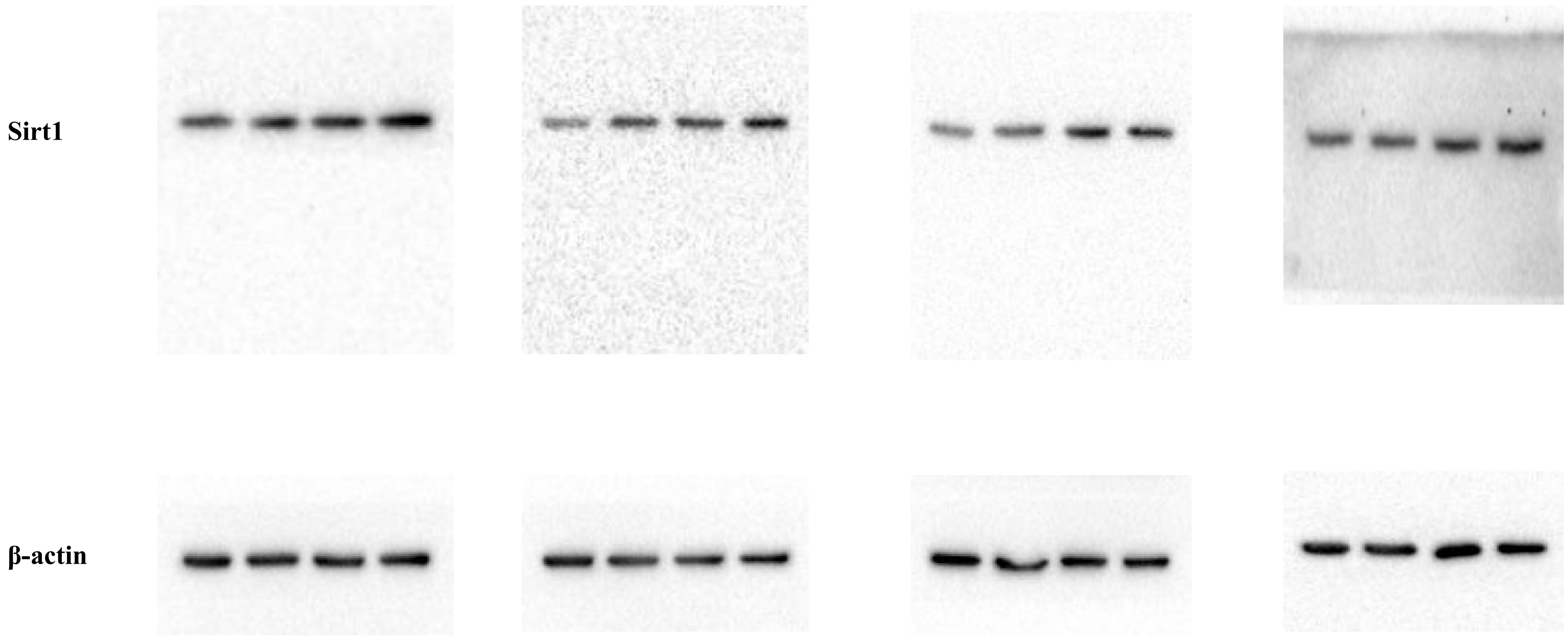
Sirt1
β-actin

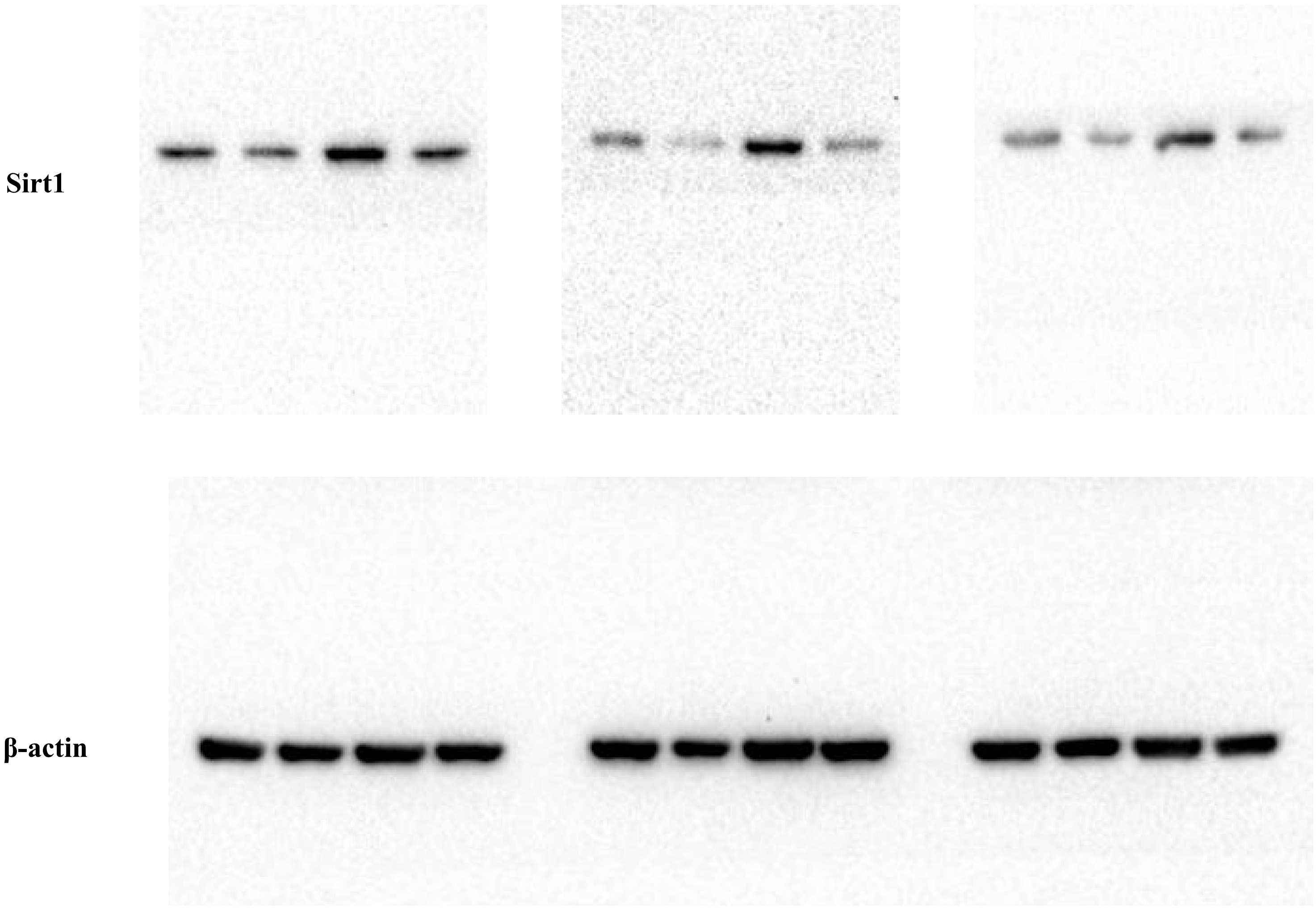

Sirt1
β-actin

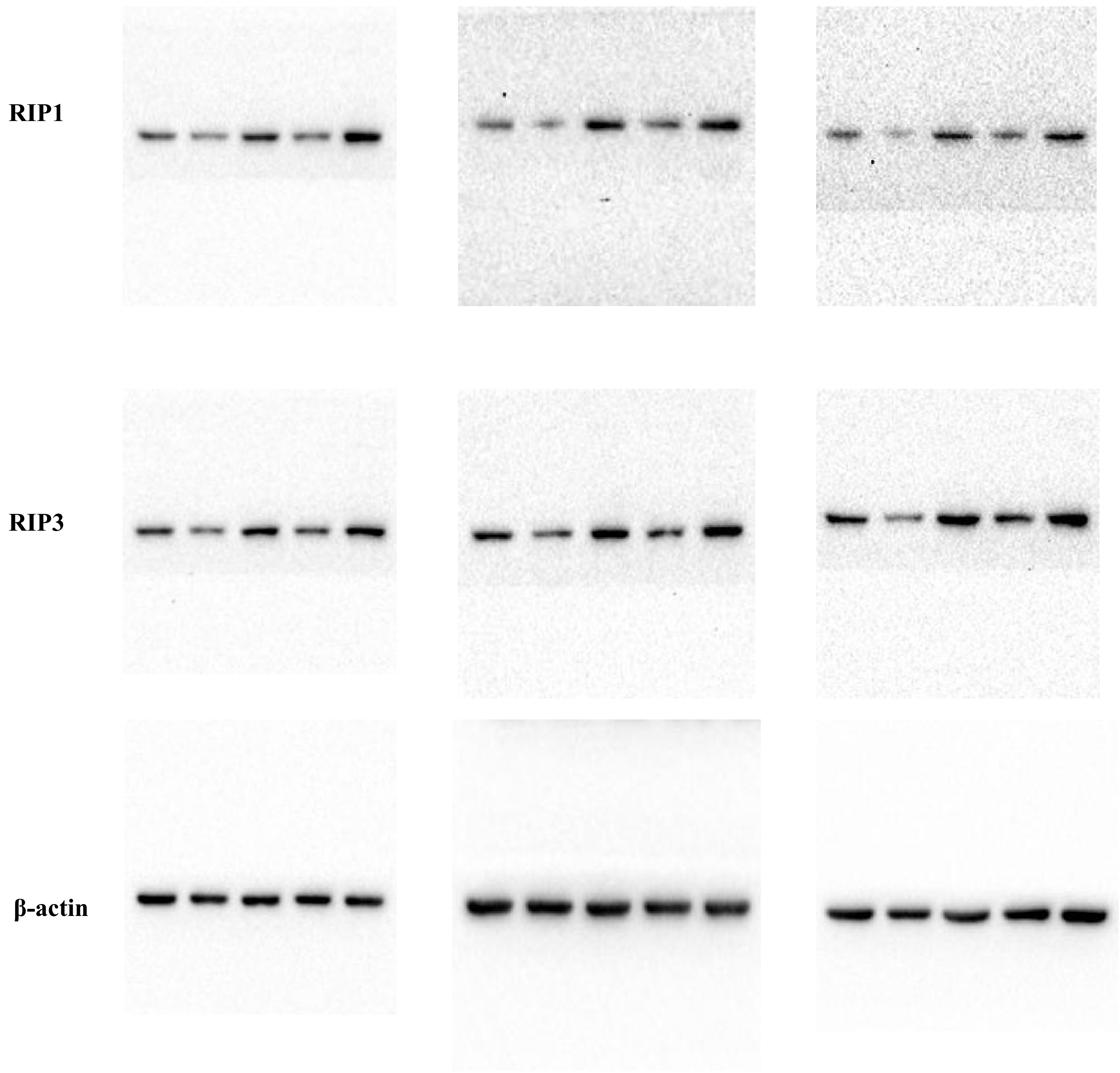
RIP1
RIP3
β-actin

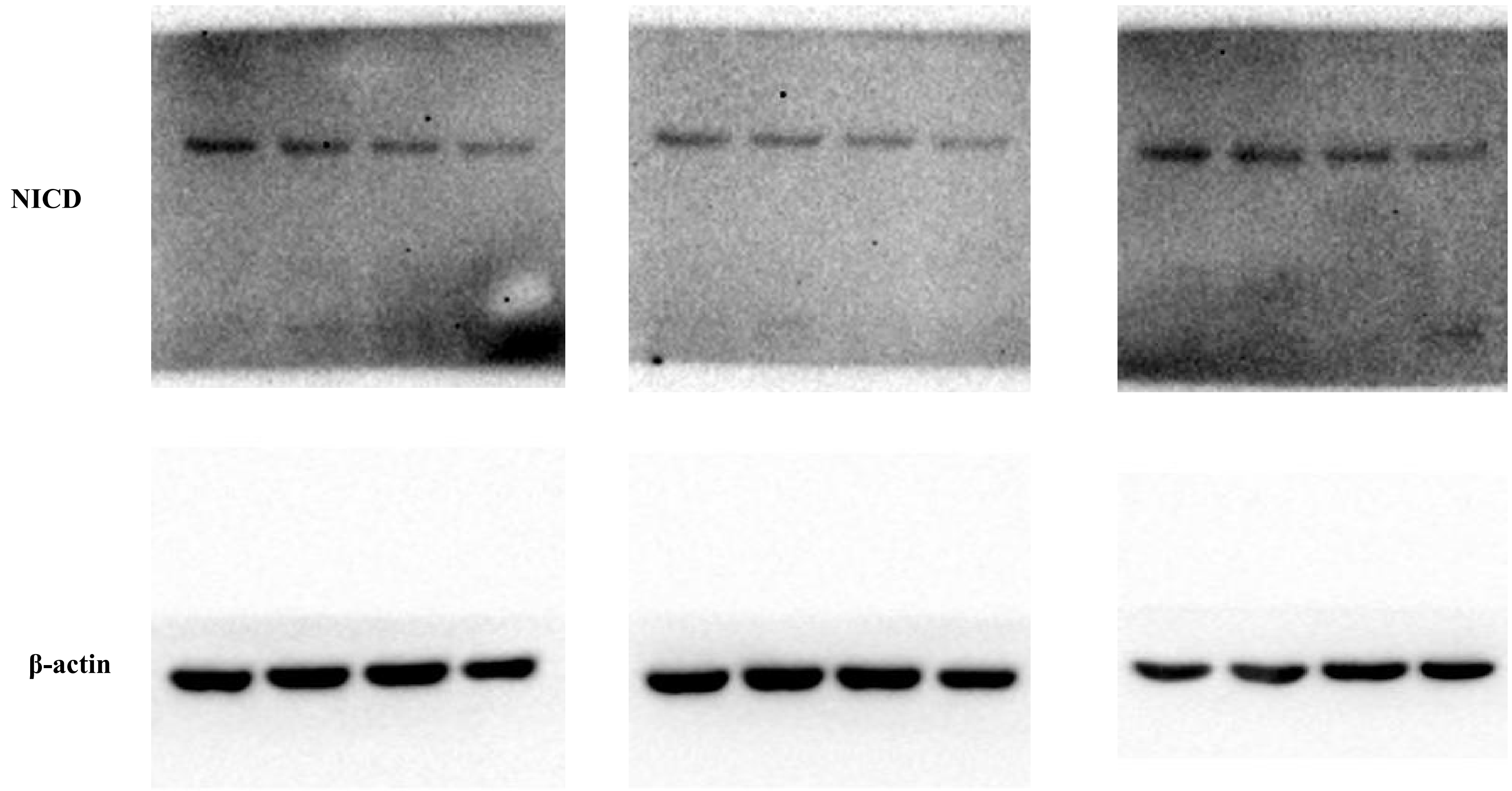
NICD
β-actin

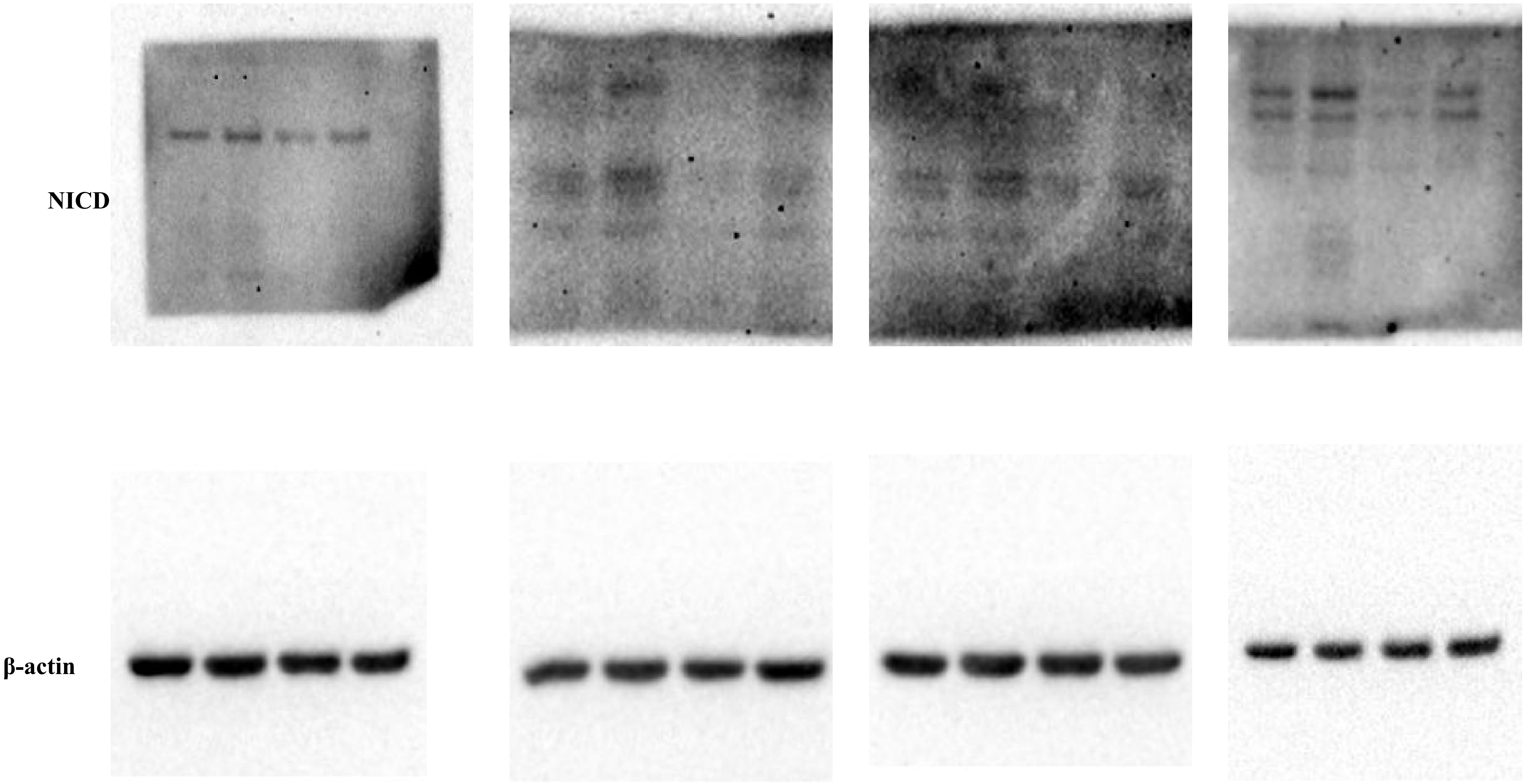
NICD
β-actin

NICD

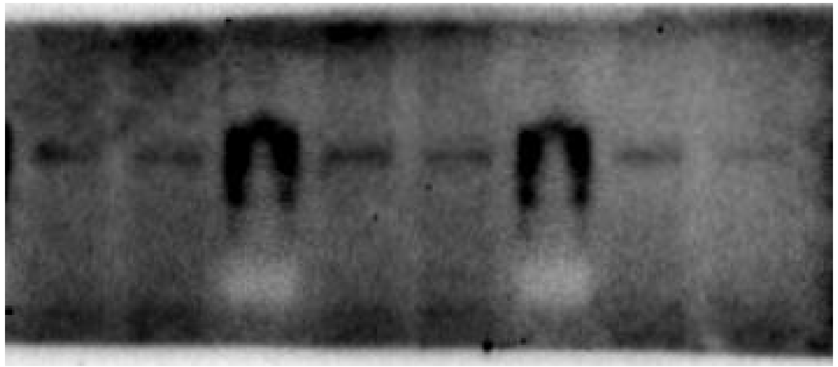

β-actin

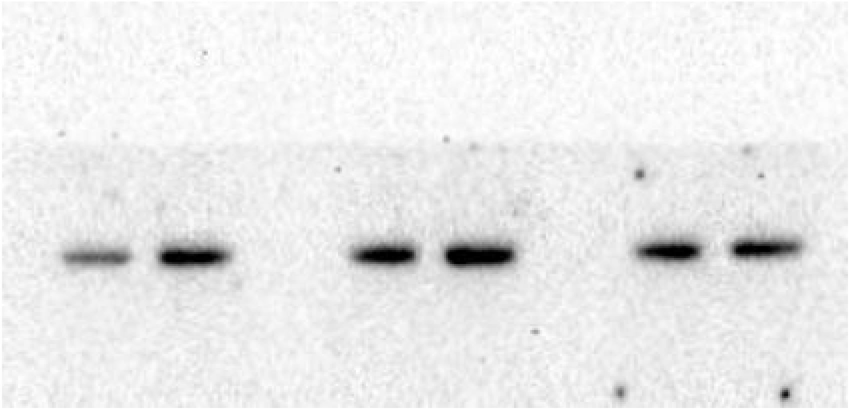

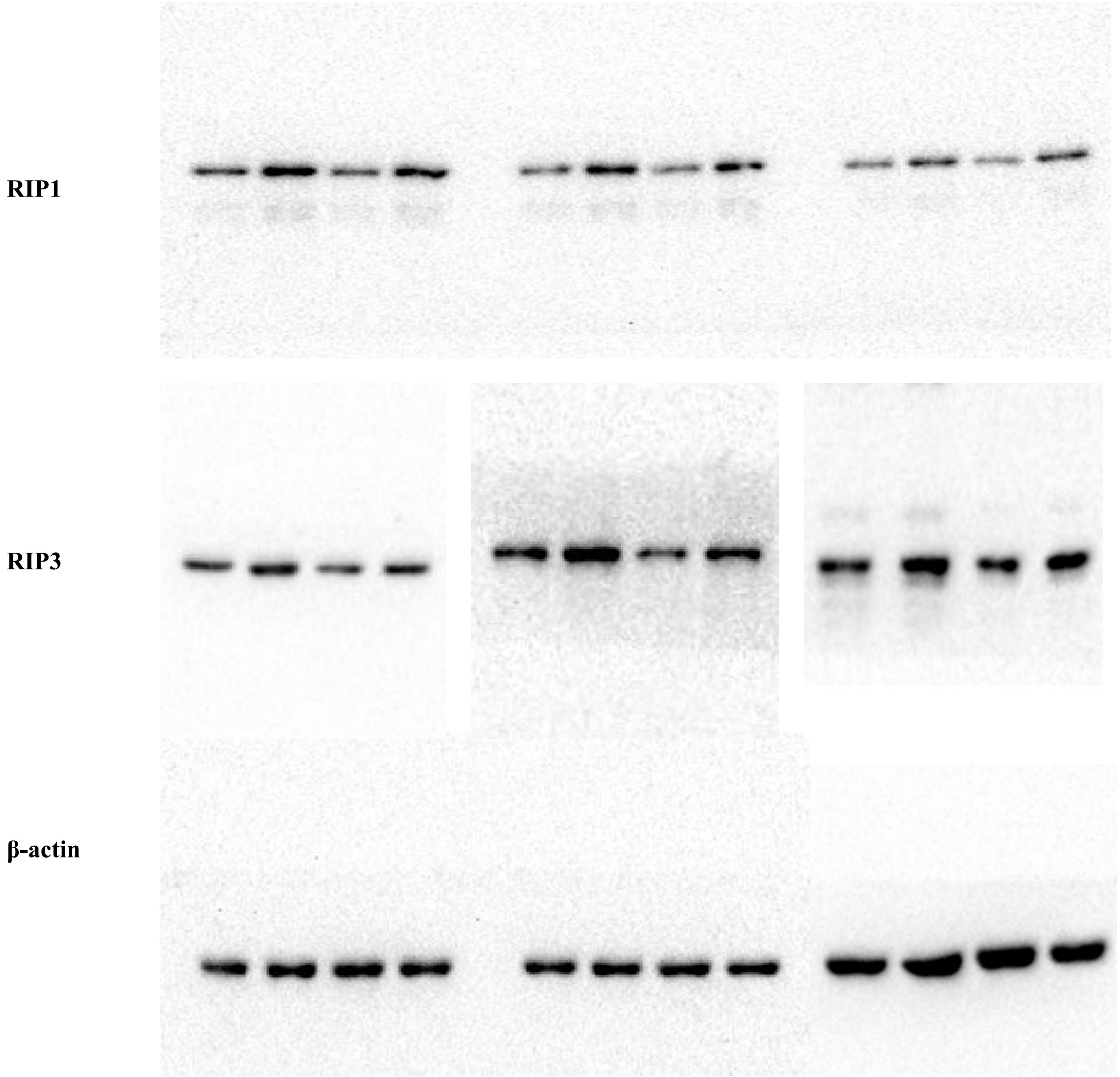
RIP1
RIP3
β-actin
